# THE 12 DS: AN UPDATE TO EDWARDS’ AND RAY’S REASONS FOR NONPARENTAL CAREGIVING

**DOI:** 10.1093/geroni/igad104.3070

**Published:** 2023-12-21

**Authors:** Acacia Lopez, Danielle Nadorff

**Affiliations:** Mississippi State University, Mississippi State, Mississippi, United States; Mississippi State University, Mississippi State, Mississippi, United States

## Abstract

This study examined the prevalence of the “Nine D’s,” a framework developed by Edwards and Ray for understanding the heterogeneity of reasons for which grandparents assume care of grandchildren (i.e., Death, Disease, Detention, Divorce, Departure, Drugs, Desertion, Delivery, Deployment) within a contemporary sample. Using a nationwide sample (N = 427) of custodial grandparents and foster parents, caregivers were asked their reason for assuming care of the grandchild or foster child within their care. The results of the study suggest that the Nine D’s are a useful framework, but fail to capture many of the reasons for assuming care. Three new themes—Dollars, Duty, and Daily Grind—were identified and are applicable to both grandfamilies and foster families. These themes represent different motivations for assuming care and provide insight into the social structures which may act as barriers to family formation. This study provides a foundation for future research examining the impact of assumed care by non-parental attachment figures on the health and well-being of both grandchildren and foster children.

